# Correction: Evaluation of mAb 2C5-modified dendrimer-based micelles for the co-delivery of siRNA and chemotherapeutic drug in xenograft mice model

**DOI:** 10.1007/s13346-024-01601-1

**Published:** 2024-04-18

**Authors:** Satya Siva Kishan Yalamarty, Nina Filipczak, Tanvi Pathrikar, Colin Cotter, Janaína Artem Ataide, Ed Luther, Swarali Paranjape, Vladimir Torchilin

**Affiliations:** 1 Center for Pharmaceutical Biotechnology and Nanomedicine, Northeastern University, Boston, MA 02115 USA; 2 Department of Pharmaceutical Sciences, Northeastern University, Boston, MA 02115 USA; 3 Department of Chemical Engineering, Northeastern University, Boston, MA 02115 USA; 4 Faculty of Pharmaceutical Sciences, University of Campinas (UNICAMP), Campinas, SP 13083-871 Brazil

**Correction to: Drug Delivery and Translational Research** 10.1007/s13346-024-01562-5

In the original online version of this article there were treatment groups missing in Fig. 6. Following is the corrected Fig. 6. The original article was corrected:

**Fig. 6:**
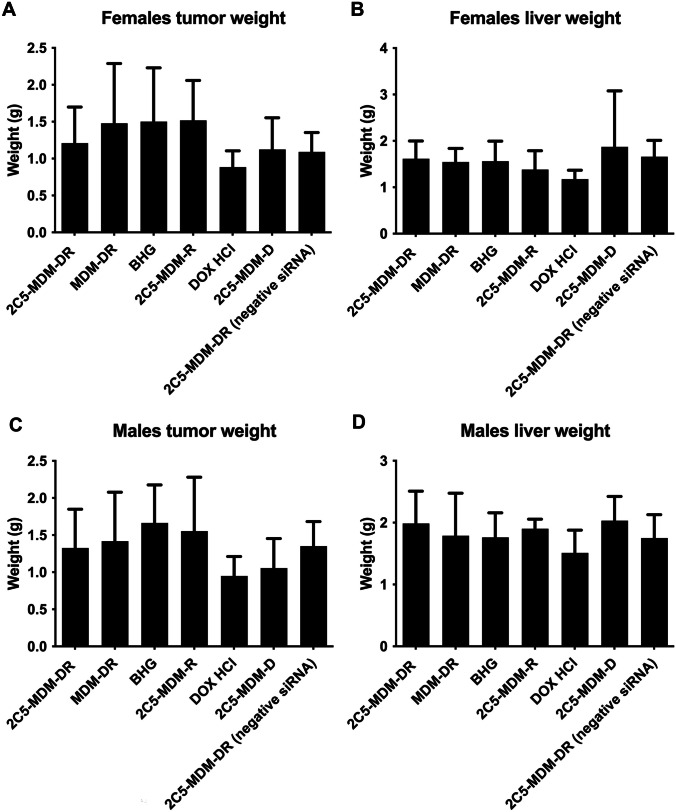
Weights of harvested tumors and livers in female and male mice. The tumors weights show the effectiveness of the treatment and the weights of the liver show the safety of the treatment, **A** Tumor weights in female mice, **B** Liver weights in female mice, **C** Tumor weights in male mice, **D** Liver weights in male mice

